# Generation of KCL040 clinical grade human embryonic stem cell line

**DOI:** 10.1016/j.scr.2015.12.035

**Published:** 2016-01

**Authors:** Laureen Jacquet, Victoria Wood, Neli Kadeva, Glenda Cornwell, Stefano Codognotto, Emma Stephenson, Dusko Ilic

**Affiliations:** Stem Cell Laboratories, Division of Women's Health, Faculty of Life Sciences and Medicine, King's College London and Assisted Conception Unit, Guys' Hospital, London, United Kingdom

## Abstract

The KCL040 human embryonic stem cell line was derived from a normal healthy blastocyst donated for research. The ICM was isolated using laser microsurgery and plated on γ-irradiated human foreskin fibroblasts. Both the derivation and cell line propagation were performed in an animal product-free environment and under current Good Manufacturing Practice (cGMP) standards. Pluripotent state and differentiation potential were confirmed by in vitro assays.

## Resource table

**Table:** 

Name of stem cell line	KCL040
Institution	King's College London, London UK
Derivation team	Neli Kadeva, Victoria Wood, Glenda Cornwell, Stefano Codognotto, Emma Stephenson
Contact person and email	Dusko Ilic, email: dusko.ilic@kcl.ac.uk
Date archived/stock date	Feb 03, 2012
Type of resource	Biological reagent: cell line
Sub-type	Human pluripotent stem cell line
Origin	Human embryo
Key marker expression	Pluripotent stem cell markers: NANOG, OCT4, TRA-1-60, TRA-1-81, alkaline phosphatase (AP) activity
Authentication	Identity and purity of line confirmed
Link to related literature (direct URL links and full references)	1) Jacquet, L., Stephenson, E., Collins, R., Patel, H., Trussler, J., Al-Bedaery, R., Renwick, P., Ogilvie, C., Vaughan, R., Ilic, D., 2013. Strategy for the creation of clinical grade hESC line banks that HLA-match a target population. EMBO Mol. Med. 5 (1), 10–17. doi: 10.1002/emmm.201201973 http://www.ncbi.nlm.nih.gov/pubmed/23161805 2) Canham, A., Van Deusen, A., Brison, D.R., De Sousa, P., Downie, J., Devito, L., Hewitt, Z.A., Ilic, D., Kimber, S.J., Moore, H.D., Murray, H., Kunath, T., 2015. The molecular karyotype of 25 clinical-grade human embryonic stem cells lines. Sci. Rep*.* 5, 17258. doi: 10.1038/srep17258 http://www.ncbi.nlm.nih.gov/pubmed/26607962 3) Ilic, D., Stephenson, E., Wood, V., Jacquet, L., Stevenson, D., Petrova, A., Kadeva, N., Codognotto, S., Patel, H., Semple, M., Cornwell, G., Ogilvie, C., Braude, P., 2012. Derivation and feeder-free propagation of human embryonic stem cells under xeno-free conditions. Cytotherapy. 14 (1), 122–128. doi: 10.3109/14653249.2011.623692 http://www.ncbi.nlm.nih.gov/pubmed/22029654 4) Stephenson, E., Jacquet, L., Miere, C., Wood, V., Kadeva, N., Cornwell, G., Codognotto, S., Dajani, Y., Braude, P., Ilic, D., 2012. Derivation and propagation of human embryonic stem cell lines from frozen embryos in an animal product-free environment. Nat. Protoc. 7 (7), 1366–1381. doi: 10.1038/nprot.2012.080 http://www.ncbi.nlm.nih.gov/pubmed/22722371
Information in public databases	KCL040 is a National Institutes of Health (NIH) registered hESC line NIH Registration Number: NIHhESC-14-0272 http://grants.nih.gov/stem_cells/registry/current.htm?id=678
Ethics	The hESC line KCL040 is derived under license from the UK Human Fertilisation and Embryology Authority (research licence numbers: R0075 and R0133) and also has local ethical approval (UK National Health Service Research Ethics Committee Reference: 06/Q0702/90). Informed consent was obtained from all subjects and the experiments conformed to the principles set out in the WMA Declaration of Helsinki and the NIH Belmont Report. No financial inducements are offered for donation.

## Resource details

**Table:** 

Consent signed	Sep 03, 2010
Embryo thawed	Jan 17, 2012
UK Stem Cell Bank Deposit Approval	Reference: SCSC12-37
Sex	Female 46, XX
Grade	Clinical
Disease status	Healthy/Unaffected
Karyotype (aCGH)	Reduced copy number at 5q13.2 (69,705,561–70,388,844).
SNP Array	Copy-neutral loss of heterozygosity (CN-LOH) at 2q11.1–11.2 (94,871,756–98,412,364), gain at 12p11.21 (31,116,366–31,248,444), loss at 16p11.2 (32,491,547–33,993,220) (Canham et al., 2015)
DNA fingerprint	Allele sizes (in bp) of 16 microsatellite markers specific for chromosomes 13, 18 and 21 (Jacquet et al., 2013)
HLA typing	HLA-A 03, 24; B 07, 15; Bw 4, 6; C 03, 07; DRB1 04, 15; DRB4 01; DRB5 01; DQB1 03, 06 (Jacquet et al., 2015, Canham et al., 2015)
Viability testing	Pass
Mycoplasma	Negative
Sterility	Pass
Pluripotent markers (immunostaining) (Fig. 1)	NANOG, OCT4, TRA-1-60, TRA-1-81, AP activity
Three germ layers differentiation in vitro (immunostaining) (Fig. 2)	Endoderm: AFP Ectoderm: TUBB3 (tubulin, beta 3 class III) Mesoderm: ACTA2 (actin, alpha 2, smooth muscle)
Sibling lines available	No

**Fig. 1:**
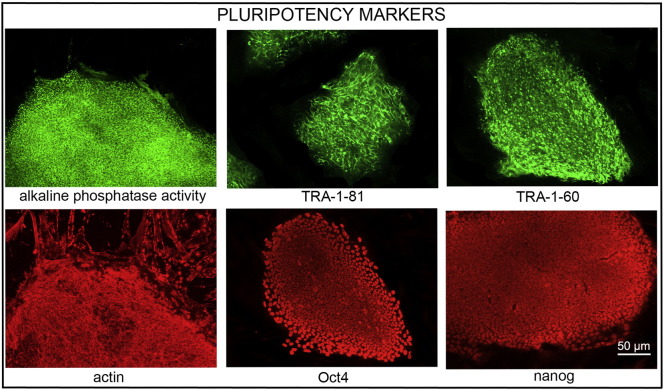
Expression of pluripotency markers. Pluripotency is confirmed by immunostaining (Oct4, Nanog, TRA-1-60, TRA-1-81) and alkaline phosphatase (AP) activity assay. Actin stress fibers, visualized with rhodamine-phalloidin (red), are present in both feeders and hES cell colonies, whereas AP activity (green) is detected only in hES cells. Scale bar, 50 μm.

**Fig. 2:**
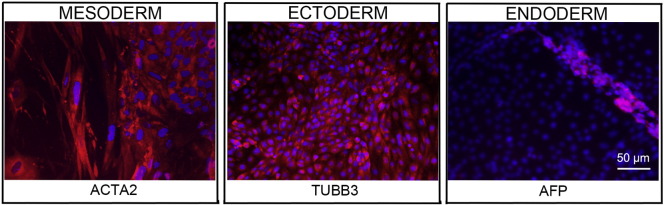
Differentiation of three germ layers in vitro is confirmed by detection of markers: smooth muscle actin (red) for mesoderm, β-III tubulin (red) for ectoderm and α-fetoprotein (red) for endoderm. Nuclei are visualized with Hoechst 33,342 (blue). Scale bar, 50 μm.

We generated KCL040 clinical grade hESC line following protocols, established previously (Ilic et al., 2012, Stephenson et al., 2012), and now adapted to cGMP conditions. The expression of the pluripotency markers was tested after freeze/thaw cycle (Fig. 1). Differentiation potential into three germ layers was verified in vitro (Fig. 2).

Molecular karyotyping using array comparative genomic hybridization aCGH identified reduced copy number at 5q13.2 (69,705,561–70,388,844). The imbalance was not called by software. Whole-genome single nucleotide polymorphism (SNP) array analysis detected CN-LOH at 2q11.1–11.2 (94,871,756–98,412,364), gain at 12p11.21 (31,116,366–31,248,444), loss at 16p11.2 (32,491,547–33,993,220) (Canham et al., 2015).

This CN-LOH at 2q11.1–11.2 contains multiple genes: *TEKT4*, *MAL*, *MRPS5*, *ZNF514*, *ZNF2*, *PROM2*, *KCNIP3*, *FAHD2A*, *TRIM43*, *ANKRD36C*, *GPAT2*, *ADRA2B*, *ASTL*, *DUSP2*, *STARD7*, *TMEM127*, *CIAO1*, *SNRNP200*, *ITPRIPL1*, *NCAPH*, *NEURL3*, *ARID5A*, *KANSL3*, *FER1L5*, *LMAN2L*, *CNNM4*, *CNNM3*, *ANKRD23*, *ANKRD39*, *SEMA4C*, *FAM178B*, *FAHD2B*, *ANKRD36*, *ANKRD36B*, *COX5B*, *ACTR1B*, *ZAP70*, *TMEM131*, *VWA3B*, and *CNGA3*. Genetic size of this interstitial CN-LOH is relatively small and the double recombination event required to this to happen would be difficult to explain (Kryh et al., 2011, O'Keefe et al., 2010). Therefore, it is unlikely that is acquired (Canham et al., 2015).

The gain on chromosome 12p11.21 was also found in KCL033. The region contains no genes and it has been also reported in at least 14 submissions at Database of Genomic Variants (DGV; http://dgv.tcag.ca), which has collected structural variations in more than 14,000 healthy individuals from worldwide population (Macdonald et al., 2014). Estimated frequency in the human population is 4.70% (Canham et al., 2015).

The loss at 16p11.2 contains three related genes *TP53TG3*, *TP53TG3C*, and *TP53TG3B* and it was reported previously in healthy population (Shaikh et al., 2009, de Smith et al., 2007). Estimated frequency in the human population is 5.14% (Canham et al., 2015).

The KCL040 line was negative for Human Immunodeficiency Virus 1 (HIV1), Hepatitis B (HepB, HCB), C Virus (HepC, HCV), Cytomegalovirus (CMV) and Epstein–Barr Virus (EBV) by PCR. Mycoplasma was also not detected.

We also generated research grade of KCL040 line that is adapted to feeder-free conditions.

## Materials and methods

### Consenting process

We distribute Patient Information Sheet (PIS) and consent form to the in vitro fertilization (IVF) patients if they opted to donate to research embryos that were stored for 5 or 10 years. They mail signed consent back to us and that might be months after the PIS and consent were mailed to them. If in meantime new versions of PIS/consent are implemented, we do not send these to the patients or ask them to re-sign; the whole process is done with the version that was given them initially. The PIS/consent documents (FRO-V.8) were created on Mar. 11, 2010. HFEA Code of Practice that was in effect at the time of document creation: Edition 8 — R.1 (http://www.hfea.gov.uk/2999.html). The donor couple signed the consent on Sep. 03, 2010. HFEA Code of Practice that was in effect at the time of donor signature: Edition 8 — R.2. HFEA Code of Practice Edition 8 — R.1 was in effect: Oct. 01 2009–Apr. 06, 2010, whereas 8 — R.2 was in effect: Apr. 07, 2010–Apr. 06, 2011.

### Embryo culture and micromanipulation

Embryo culture and laser-assisted dissection of inner cell mass (ICM) were carried out as previously described in details (Ilic et al., 2012, Stephenson et al., 2012). The cellular area containing the ICM was then washed and transferred to plates containing mitotically inactivated human neonatal foreskin fibroblasts (HFF).

### Cell culture

ICM plated on mitotically inactivated HFF was cultured as described (Ilic et al., 2012, Stephenson et al., 2012). TE cells were removed mechanically from outgrowth (Ilic et al., 2007, Ilic et al., 2010). hESC colonies were expanded and cryopreserved at the third passage.

### Viability test

Straws with the earliest frozen passage (p.2–3) are thawed and new colonies are counted three days later. These colonies are then expanded up to passage 8, at which point cells were part frozen and part subjected to standard battery of tests (pluripotency markers, in vitro and in vivo differentiation capability, genetics, sterility, mycoplasma).

### Pluripotency

Pluripotency in vitro was assessed using two different techniques: enzymatic activity assay [alkaline phosphatase (AP) assay] and immunostaining as described (Ilic et al., 2012, Stephenson et al., 2012).

### Differentiation

Spontaneous differentiation into three germ layers was assessed in vitro and in vivo as described (Petrova et al., 2014, Stephenson et al., 2012). Targeted differentiation in cardiomyocytes followed the protocols described earlier (Jacquet et al., 2015, Laflamme et al., 2007).

### Genotyping

DNA was extracted from hESC cultures using a Chemagen DNA extraction robot according to the manufacturer's instructions. Amplification of polymorphic microsatellite markers was carried out as described (Ilic et al., 2012). Allele sizes were recorded to give a unique fingerprint of each cell line.

### Array comparative genomic hybridization (aCGH)

aCGH was performed as described in details (Ilic et al., 2012).

### Whole-genome single nucleotide polymorphism (SNP) array

SNP array was performed as described in details (Canham et al., 2015).

### HLA typing

HLA-A, -B and -DRB1 typing was performed with a PCR sequence-specific oligonucleotide probe (SSOP; Luminex, Austin, TX, USA) hybridization protocol at the certified Clinical Transplantation Laboratory, Guy's and St Thomas' NHS Foundation Trust and Serco Plc. (GSTS) Pathology (Guy's Hospital, London, UK) as described (Jacquet et al., 2013). HLA typing was also performed independently by other group (Canham et al., 2015).

### Special pathology

The Doctors Laboratory London (UK) tested the line for HIV1, HepB, HepC, CMV and EBV by PCR.

## Author disclosure statement

There are no competing financial interests in this study.
